# Oxyresveratrol-Loaded PLGA Nanoparticles Inhibit Oxygen Free Radical Production by Human Monocytes: Role in Nanoparticle Biocompatibility

**DOI:** 10.3390/molecules26144351

**Published:** 2021-07-18

**Authors:** Marta Donini, Salvatore Calogero Gaglio, Carlo Laudanna, Massimiliano Perduca, Stefano Dusi

**Affiliations:** 1 Department of Medicine, Section of General Pathology, University of Verona, Strada Le Grazie 8, 37134 Verona, Italy; marta.donini@univr.it (M.D.); carlo.laudanna@univr.it (C.L.); stefano.dusi@univr.it (S.D.); 2 Department of Biotechnology, University of Verona, Strada Le Grazie 15, 37134 Verona, Italy; salvatorecalogero.gaglio@univr.it

**Keywords:** oxyresveratrol, β-glucan, PLGA nanoparticles, ROS, monocytes

## Abstract

Oxyresveratrol, a polyphenol extracted from the plant *Artocarpus lakoocha* Roxb, has been reported to be an antioxidant and an oxygen-free radical scavenger. We investigated whether oxyresveratrol affects the generation of superoxide anion (O_2_^−^) by human monocytes, which are powerful reactive oxygen species (ROS) producers. We found that oxyresveratrol inhibited the O_2_^−^ production induced upon stimulation of monocytes with β-glucan, a well known fungal immune cell activator. We then investigated whether the inclusion of oxyresveratrol into nanoparticles could modulate its effects on O_2_^−^ release. We synthesized poly(lactic-co-glycolic acid) (PLGA) nanoparticles, and we assessed their effects on monocytes. We found that empty PLGA nanoparticles induced O_2_^−^ production by resting monocytes and enhanced the formation of this radical in β-glucan-stimulated monocytes. Interestingly, the insertion of oxyresveratrol into PLGA nanoparticles significantly inhibited the O_2_^−^ production elicited by unloaded nanoparticles in resting monocytes as well as the synergistic effect of nanoparticles and β-glucan. Our results indicate that oxyresveratrol is able to inhibit ROS production by activated monocytes, and its inclusion into PLGA nanoparticles mitigates the oxidative effects due to the interaction between these nanoparticles and resting monocytes. Moreover, oxyresveratrol can contrast the synergistic effects of nanoparticles with fungal agents that could be present in the patient tissues. Therefore, oxyresveratrol is a natural compound able to make PLGA nanoparticles more biocompatible.

## 1. Introduction

Monocytes are a heterogeneous cell population initiating and propagating the immune response to pathogenic microorganisms. These cells circulate in the bloodstream and pass into the tissues where they may differentiate into resident macrophages. During inflammation, the quantity of extravasated monocytes increases to amplify the local immune response through cytokine release and phagocytosis of foreign microorganisms [1]. Upon pathogen uptake, monocytes produce reactive oxygen species (ROS) [2]. The main source of ROS in monocytes is the NOX2 NADPH oxidase, a multicomponent enzyme which transfers electrons to molecular oxygen to generate superoxide anion (O_2_^−^) [3,4]. ROS play an important role in defenses against infections [3,4,5], but they may also damage the cells by causing oxidative stress, which is responsible for several diseases, such as cancer, hypertension and neurological disorders [6].

Oxyresveratrol, a natural polyphenol extracted from the plant *Artocarpus lakoocha* Roxb, has been reported to be a good antioxidant, equipped with ROS scavenger activity [7]. In particular oxyresveratrol protects neurons [8,9,10,11], human lens epithelial cells [12] and hepatocytes [13], reduces adverse effects of nicotine [14] and mitigates DNA damage [15] by mechanisms involving inhibition of ROS generation. Moreover, oxyresveratrol is able to decrease the production of the free radical nitric oxide, which can cause cell injury in many inflammatory diseases, in RAW264.7 murine macrophage cell line [16,17]. However, as far as we know, no investigations have been performed to assess whether oxyresveratrol inhibits ROS production in human leucocytes, which are able to produce high amounts of oxygen radicals when activated by pathogenic microorganisms [3,4,5]. Therefore we examined whether a mechanism by which oxyresveratrol blunts the oxidative damage would reside in the inhibition of ROS production by human monocytes.

Recently several nanoparticles, including lipid-based [18], pterostilbene-loaded [19] and poly(lactic-co-glycolic acid) (PLGA) [20,21] nanostructures, have been synthesized to improve the bioavailability, solubility and retention time of various bioactive molecules in order to use them for medical purposes In fact, several molecules of natural origin, potentially useful for medical therapy, suffer from poor solubility in an aqueous environment, with a consequent low bioavailability and sometimes a very low chemical stability in this environment, decreasing the possibility of using them as active principles [22]. However, the insertion of these molecules into nanoparticles of different origins increases all the parameters that are essential to a good drug to have the expected efficacy, including its half-life and interaction with target cells [18,19,20,23]. Some examples of such enhanced features are given by genistein, an isoflavonoid with tyrosine kinase inhibitor activity that useful in cancer therapy, whose effectiveness has been increased by the encapsulation in polymeric nanomaterials [24]; resveratrol, a natural polyphenol with a broad spectrum of pharmacological activities showing enhanced action against cardiovascular diseases when embedded in nanoformulations [25]; quercetin, a plant flavonoid whose efficacy as an antitumoral molecule is cancelled by its low solubility and consequent low bioavailability but is greatly enhanced by using nanocarriers [26]; and curcumin, another natural phenolic compound derived from the rhizome of *Curcuma longa*, proven to have many pharmacological activities and whose embedding in polymeric nanoparticles increases its oral bioavailability [27]. In particular, biodegradable polymeric nanoparticles, such as PLGA, polylactic acid (PLA), chitosan and gelatin, have been extensively used to ameliorate the therapeutic effect of several soluble/insoluble drugs because they show a very low or absent toxicity and are degraded by cells into nontoxic compounds, with a decreased risk of unwanted side effects for patients undergoing this type of therapy [21]. These nanostructures have been exploited to successfully encapsulate bioactive molecules useful for the treatment of several diseases like cancer, AIDS, diabetes, malaria, prion disease and tuberculosis [21].

However, upon administration to patients, nanoparticles interact with the cells of the immune system which can react against these materials by producing proinflammatory mediators which lead to adverse events, such as inflammation, allergy or oxidative damage [28,29,30]. In particular it has been reported that the exposure of phagocytic cells to some nanostructures, including polymeric nanoparticles, induces ROS formation and causes inflammation [31,32,33,34]. Therefore it is important to carefully evaluate the impact of nanomaterials on immune cells before using these facilities as therapeutic means. Among the various polymers used to produce nanoparticles, PLGA has received broad interest because it is biocompatible, biodegradable and approved for human therapies both by the Food and Drug Administration (FDA) and the European Medicines Agency (EMA) [35,36,37]. So we investigated the effects of unloaded PLGA nanoparticles on ROS formation by human monocytes and we examined whether the insertion of oxyresveratrol into these particles would modulate its action on ROS generation by these cells.

## 2. Results

### 2.1. Characterization of PLGA Nanoparticles

Empty (PLGA) and oxyresveratrol-loaded PLGA nanoparticles (PLGA–Oxy) have been prepared following a previously used protocol [38]: Therefore, particle size and ζ-potential were completely comparable to those previously published by our group. To investigate the uptake of PLGA nanoparticles by monocytes we prepared fluorescence-responsive rhodamine B-loaded PLGA nanoparticles (PLGA–Rhod). As illustrated in Table 1, we were able to obtain PLGA nanoparticles embedding the dye molecule showing a hydrodynamic radius only slightly higher in comparison to PLGA and PLGA–Oxy, with an average size of 209.2 ± 0.6. The size obtained was comparable to data previously published by other research groups when similar synthesis conditions were used [39].

The encapsulation of the huge amount of rhodamine B necessary to be revealed by fluorescence microscopy affected the nanoparticles size, with an increase of the gyration radius of about 40 nm; nevertheless, the size-distribution showed a monodisperse trend, as is demonstrated by the low PDI value exhibited by all the different preparations. The ζ-potential measured at pH 7.5 appeared to be definitely more negative (−18.2 ± 1.4 mV) if compared to the PLGA–Oxy (−7.1 ± 0.5 mV) and PLGA nanoparticles (−9.6 ± 0.4 mV). Therefore, all the different nanoparticles used in this study, including the dye-loaded ones, exhibited ζ-potential values that were consistent with a good colloidal stability.

The presence of the dye in the PLGA nanoformulations was assessed by fluorescence spectroscopy. Figure S1 shows the emission pattern of PLGA–Rhod: you can see that upon excitation at 555 nm dye-loaded nanoparticles exhibited the fluorescence pattern typical of the free dye.

The amount of encapsulated dye was calculated by dissolving the PLGA–Rhod in dimethyl sulfoxide (DMSO) and inserting the values obtained by the spectrophotometric analysis in the calibration line equation. We obtained nanoparticles carrying a total dye amount of 12.18 µg of rhodamine B per mg of PLGA with an encapsulation efficiency of 48.97% ± 0.33.

### 2.2. Oxyresveratrol Inhibited the O_2_^−^ Production by Human Monocytes

The interaction between monocytes and pathogenic microorganisms induces the production of ROS which can have tissue damaging effects [3,6]. We then investigated whether oxyresveratrol would be able to decrease the ROS production elicited by stimulation of monocytes with β-glucan, a yeast-derived molecule able to activate the immune cells [40,41].

**Figure 1:**
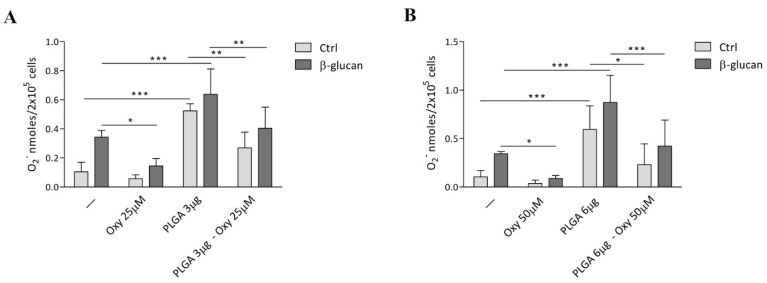
Effects of free or PLGA nanoparticle-conjugated oxyresveratrol on O_2_^−^ production by human monocytes. Monocytes were treated for 18 h with 25 μM (panel (**A**)) and 50 μM (panel (**B**)) oxyresveratrol free (Oxy) or loaded on PLGA nanoparticles (PLGA-Oxy), as well as with equivalent quantities of unloaded PLGA particles (PLGA). All the treatments were conducted in the absence (Ctrl) or presence of 5 μg/mL β-glucan. The O_2_^−^ production was evaluated by cytochrome c reduction. The results are expressed as the mean value ±SD of four independent experiments. * *p* < 0.05, ** *p* < 0.01 *** *p* < 0.001 by two way ANOVA followed by Bonferroni post-test.

For this purpose, human blood monocytes were treated with 25 μM and 50 μM free oxyresveratrol (Oxy), both in the absence (Ctrl) and the presence of 5 μg/mL β-glucan (Figure 1). After an 18 h incubation the O_2_^−^ production was analyzed by cytochrome c reduction test. Figure 1 shows that, at the above mentioned doses, oxyresveratrol significantly decreased the β-glucan-induced release of O_2_^−^ by monocytes. Adding higher doses of oxyresveratrol (100 μM) did not lead to further inhibition of ROS production (results not shown).

### 2.3. Incorporation of Oxyresveratrol into PLGA Nanoparticles Inhibited Their Ability to Induce O_2_^−^ Production in Human Monocytes

We then wondered whether the insertion of oxyresveratrol into PLGA nanoparticles could influence the effects of this polyphenol on ROS production by monocytes. First of all we checked whether unloaded PLGA particles prepared in our laboratory would affect the O_2_^−^ generation by human monocytes. Figure 1 shows that the incubation of resting monocytes with 3 μg (panel A) or 6 μg (panel B) of bare PLGA particles triggered O_2_^−^ production. Moreover, unloaded PLGA particles significantly enhanced the O_2_^−^ generation by β-glucan-stimulated monocytes (Figure 1 panel A and B). These results indicate that the nanoparticles synthesized in our laboratory induce per se ROS production by human monocytes, an event very probably triggered by activation of these cells during the uptake of the nanoparticles [2]. Interestingly, PLGA nanoparticles enhanced the ROS generation elicited by β-glucan, indicating a synergistic action between the nanoparticles and the fungal derivative in the stimulation of O_2_^−^ generation. Figure 1 also shows the results of the challenge of both resting and β-glucan stimulated monocytes with 3 μg of PLGA nanoparticles loaded with 25 μM oxyresveratrol (panel A) or 6 μg of PLGA nanoparticles loaded with 50 μM oxyresveratrol (panel B). The figure illustrates that oxyresveratrol encapsulation into PLGA nanoparticles significantly inhibited both the O_2_^−^ production caused by unloaded nanoparticles in resting monocytes and the synergistic effect of these particles and β-glucan. These findings indicate that oxyresveratrol also maintains its antioxidant properties once conjugated to PLGA nanoparticles and can reduce unwanted effects of these particles on ROS release by monocytes.

### 2.4. Evaluation of Oxyresveratrol and PLGA Nanoparticle Toxicity on Human Monocytes

**Figure 2:**
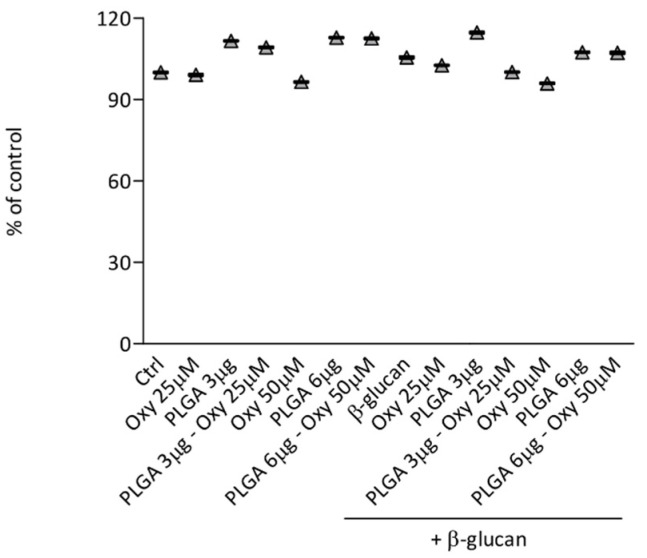
Effects of free oxyresveratrol and oxyresveratrol-bearing PLGA nanoparticles on the viability of monocytes. Monocytes were treated with the indicated doses of free oxyresveratrol (Oxy), unloaded PLGA nanoparticles (PLGA) or oxyresveratrol-loaded PLGA particles (PLGA-Oxy) for 18 h in the absence (Ctrl) or presence of 5 μg/mL β-glucan, and then incubated for 1 h with WST. The values are expressed as the percentage of WST reduction relative to untreated cells (designated as 100%). Data are means ± SD of four experiments.

To exclude that our results on ROS production could be due to toxic effects we examined whether oxyresveratrol free or inserted into PLGA nanoparticles would affect the viability of human monocytes. For this purpose, monocytes were treated with free oxyresveratrol (Oxy), unloaded PLGA or PLGA–Oxy in the absence or presence of β-glucan as in the experiments depicted in Figure 1, and cell viability was assessed using the Cell Proliferation Reagent WST-1 assay. Figure 2 shows that under these experimental conditions the viability of monocytes was not or was only slightly altered.

### 2.5. PLGA Nanoparticles’ Uptake by Human Monocytes

**Figure 3:**
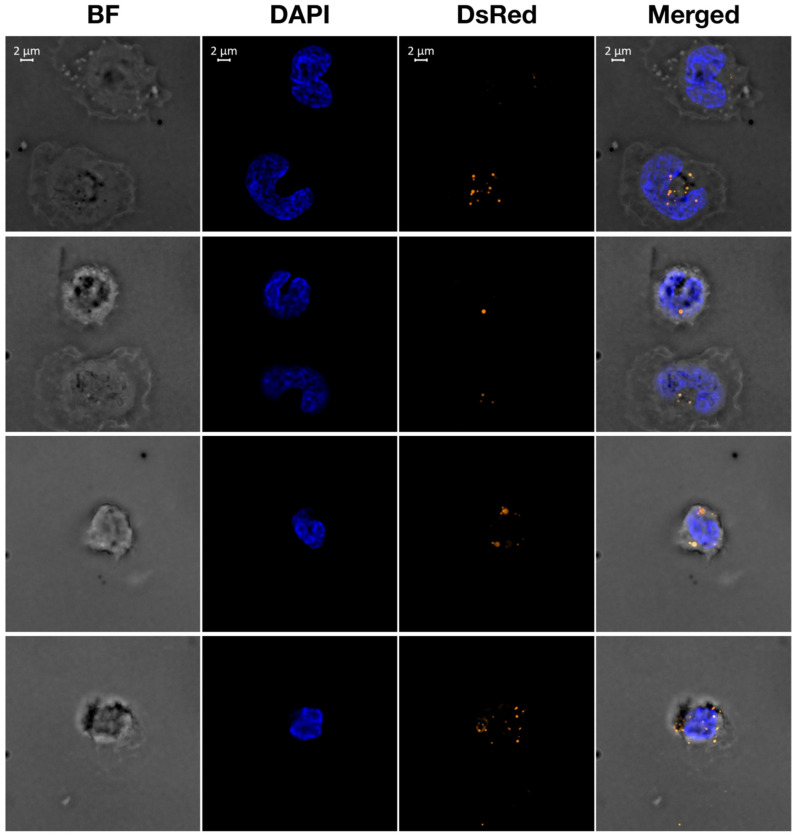
Internalization of PLGA nanoparticles by human primary monocytes. Shown are four representative fields (top to bottom rows) illustrating adherent human primary monocytes treated with rhodamine B-loaded PLGA nanoparticles for 18 h and co-stained with DAPI. From left to right, bright field (BF), DAPI and DsRed (rhodamine B) individual channels are shown. On the far right, merged channels are shown. Scale bar is 2 µm.

We then investigated whether in our experimental conditions human monocytes were able to internalize the PLGA nanoparticles synthesized and characterized in our laboratory. For this purpose, monocytes were incubated with PLGA–Rhod. Particle cellular uptake was visualized by wide-field fluorescence deconvolution microscopy at different time points. This analysis revealed that after 18 h PLGA–Rhod were efficiently internalized by human monocytes (Figure 3). Three-dimensional scanning reconstruction showed that the particles were, indeed, localized inside the cells (not shown).

## 3. Discussion

Oxyresveratrol, a natural polyphenol derived from *Artocarpus lakoocha* Roxb, has been reported to be endowed with antioxidant activity in several experimental models [7,8,9,10,11,12,13,14,15,16,17,42]. In this paper we show the results of investigations aimed at clarifying whether oxyresveratrol inhibits the ROS production in human monocytes. We chose these cells because they are able to produce large amounts of oxygen radicals through activation of the enzyme NOX2 NADPH oxidase [2,3,4]. These radicals have a defensive action [3], but they can also cause oxidative tissue damage during inflammation [6]. In spite of this, as far as we know no data have been reported on the effects of oxyresveratrol on ROS generation by human monocytes. Here we show that free oxyresveratrol decreased the O_2_^−^ generation by human monocytes stimulated with β-glucan, a treatment which mimics the natural interaction between pathogenic fungi and immune cells [40,41]. This finding indicates that oxyresveratrol has a protective activity against ROS produced by monocytes during the inflammatory process. Moreover, we wondered whether the effects of oxyresveratrol on β-glucan-stimulated monocytes could be modulated by its insertion into particles that could stabilize it and promote its internalization by the cells, and for this purpose we synthesized PLGA nanoparticles. Here we show that these nanoparticles undergo very efficient uptake by human monocytes, and therefore they are very effective molecular vehicles to carry oxyresveratrol inside these cells. The finding that unloaded PLGA nanoparticles induced O_2_^−^ production by monocytes was not surprising because it is well known that the exposure of phagocytic cells to some nanostructures triggers ROS formation [31,32,33]. With regard to PLGA particles, it has been shown that they can activate the production of ROS in human peripheral blood phagocytes [34]. This ROS generation is linked to the ingestion of nanoparticles by immune cells: In fact, the uptake of foreign materials by phagocytes leads to activation of NADPH oxidase, which is responsible for the production of O_2_^−^ [2]. Interestingly PLGA nanoparticles enhanced the ROS production induced by β-glucan. We do not know the mechanisms of this effect, but it might depend on a cooperation between pathways elicited during the engulfment of PLGA particles and those induced by β-glucan. This synergy is reminiscent of the event named “cell priming”, characterized by the ability of some agents, such as bacterial products, to increase the responsiveness of leukocytes to other stimuli if simultaneously or consequently added [43,44]. In this regard, it has been reported that the microbial chemoattractant N-formyl-methionyl-leucyl-phenylalanine (fMLP) synergized the ability of ORMOSIL nanoparticles to stimulate the release of cytokines by human leukocytes [45]. Moreover, the simultaneous addition of LPS and porous silicon-TiO_2_ microparticles was much more effective than incubation with LPS alone to induce IL-12 and TNF-α secretion by human DCs [46]. Therefore a synergy between nanoparticles and microbial molecules is not a so rare and unexpected event, although it is usually poorly studied by researchers dealing with nanostructures. Very importantly, here we show that encapsulation of oxyresveratrol into PLGA nanoparticles significantly blunted the ROS production activated by unloaded nanoparticles in resting monocytes as well as the synergistic effect of PLGA particles and β-glucan.

The main source of ROS in monocytes is the NOX2 NADPH oxidase, which produces O_2_^−^ [3,4]. Many natural phenolic compounds, including celastrol, apocynin, curcumin and resveratrol, have been reported to be inhibitors of various NADPH oxidase isoforms [47]. Therefore it is conceivable that the inhibitory effect of oxyresveratrol on ROS production by monocytes depends on its action on NADPH oxidase. Further investigations are required to elucidate the mechanisms by which oxyresveratrol inhibits this enzyme.

## 4. Materials and Methods

### 4.1. Materials

RPMI 1640 and low-endotoxin FBS were obtained from Lonza (Walkersville, MD, USA); flow cytometric analysis was performed using mouse anti-human antibody CD14 (M5E2) (Biolegend, San Diego, CA, USA).

PLGA (poly[DL-lactide-co-glycolide], CAS 26780-50-7), PVA (poly[vinyl alcohol], CAS 9002-89-5), acetone (1.00013), dimethyl sulfoxide (DMSO, D-5879), oxyresveratrol (91211) and β-glucan from baker’s yeast were purchased from Sigma-Aldrich (St. Louis, MO, USA).

### 4.2. Preparation of PLGA Nanoparticles

The PLGA nanoparticles embedded with oxyresveratrol used in this study were prepared as previously described [38]. Briefly, 10 mg of the 50:50 lactide–glycolide ratio PLGA polymer and 5 mM (1.22 mg) of oxyresveratrol were co-dissolved in 1 mL of organic solvent (95% acetone and 5% DMSO); the obtained organic phase was added dropwise under stirring (2000 RPM) to 10 mL of 1% polyvinyl alcohol (PVA) aqueous solution and left overnight to evaporate the organic phase. Nanoparticles were collected and washed by centrifugation (Eppendorf Centrifuge 5804R) at 4 °C for 20 min. The purified nanoparticles were re-suspended in 1 mL of phosphate buffer saline (PBS) solution pH 7.4 and stored at 4 °C. Empty PLGA nanoparticles were prepared with the above-described protocol but without the addition of oxyresveratrol to the organic phase.

Fluorescence responsive PLGA nanoparticles were prepared by loading them with rhodamine B. In details, 10 mg of PLGA and 0.041 mM (0.25 mg) of rhodamine B were dissolved in 1 mL of organic mixture (acetone:DMSO) with ratio 87:13. The following steps were the same as those described above.

### 4.3. Size and ζ-Potential Characterization

Size and ζ-potential of PLGA nanoparticles were estimated at 25 °C using a Nano Zeta Sizer ZS (ZEN3600, Malvern Instruments, Malvern, Worcestershire, UK). Samples resuspended in PBS, being used as a stock suspension, were diluted 10 times in PBS for size measurements and into 10 mM NaClO_4_ pH 7.5 for ζ-Potential measurements, to obtain a final concentration of 1 mg/mL for the nanoformulations. Data were collected in triplicate and analyzed by the ZetaSizer 7.10 software (Malvern, Worcestershire, UK).

### 4.4. Spectroscopic Studies, Encapsulation Efficiency

To assess the presence of dye molecules inside our nanoparticles the emission pattern was recorded upon excitation at 555 nm, which is a suitable excitation wavelength for rhodamine B.

To quantify the amount of the entrapped dye (encapsulation efficiency (EE)), a direct method was used: PLGA nanoparticles embedding rhodamine B were dissolved in DMSO and the obtained solutions were analyzed at 555 nm. Results were compared to a previously prepared calibration curve (Figure S2). Encapsulation efficiency was estimated using the following equation:

(1) $$\mathrm{EE}\;\left(\%\right)=\frac{OxyR_{loaded}}{OxyR_{fed}}\;\times 100$$

### 4.5. Monocytes Preparation and Culture

After written informed consent and upon approval of the ethical committee (Prot. N. 5626, 2 February 2012; Prot. n. 57182, 16 October 2019), buffy coats from the venous blood of normal healthy volunteers were obtained from the Blood Transfusion Centre of the University of Verona. Peripheral blood mononuclear cells were isolated by Ficoll-Hypaque and Percoll (GE Healthcare Life Science) density gradients and used as a source for immunomagnetic isolation of CD14 positive cells (Miltenyi Biotec GmbH, Auburn, CA, USA). The purity of CD14^+^ cells was always greater than 98%, as determined by flow cytometry.

### 4.6. Quantification of O_2_^−^ Production

Monocytes stimulated with 5 µg/mL β-glucan or not were treated with oxyresveratrol alone or encapsulated in PLGA nanoparticles, or with corresponding amounts of bare PLGA particles for 18 h, and then the O_2_^−^ release was estimated by cytochrome c reduction. Briefly, after cell culture for the required time the medium of each well was replaced with HBSS pH 7.4, containing 80 μM ferricytochrome c type III (Sigma-Aldrich, St. Louis, MO, USA), with or without 5 μg/mL of β-glucan (Sigma-Aldrich, St. Louis, MO, USA). Cytocrome c reduction was evaluated at 550 nm by using an automated microplate reader (Bioteck^®^ Instruments Inc., Winooski, VT, USA).

### 4.7. Cell Viability Evaluation

Cell viability was assessed using the Cell Proliferation Reagent WST-1 assay (Roche Diagnostics GmbH, Mannheim, Germany) according to the manufacturer’s instructions. Monocytes resting or activated with β-glucan were treated with oxyresveratrol alone or encapsulated in PLGA nanoparticles, or with corresponding amounts of unloaded PLGA particles for 18 h. After treatment, the cell supernatant was removed and 50 µL of pre-warmed fresh complete medium were added to cells and to three empty wells (blank). A 2× WST solution was freshly prepared by dilution of the 10× WST reagent in the complete medium and a volume of 50 μL was dispensed in the wells and blank. The plate was incubated for 60 min. The absorbance (OD) of the samples was measured using a Victor3 multilabel reader (PerkinElmer, Shelton, CT, USA) at 450 nm.

### 4.8. Statistical Analysis

Data are expressed as means ± SD. Statistical analyses, including two-way ANOVA followed by Bonferroni post-test, were performed with GraphPad Prism 5 (GraphPad Software, Inc., San Diego, CA, USA).

### 4.9. Immunofluorescence and Microscopy Analysis

Monocytes were seeded on cell culture chamber slide (Corning, NY, USA) and treated for 18 h with 6 μg of PLGA nanoparticles conjugated to rhodamine B. Cells were washed with PBS and fixed with 4% paraformaldehyde (Sigma-Aldrich) for 30 min at room temperature and quenched with 50 mM NH_4_Cl. After washing, the coverslips were incubated for 10 min with DAPI (Sigma-Aldrich) to stain nuclei. Images were acquired with a wide field Zeiss AxioImager Z.2 deconvolution microscopy setting (Carlo Zeiss, Germany), equipped with Colibri 7 fluorescent LED illumination, motorized 3D scanning stage and Hamamatsu ORCA-Flash4.0 V3 Digital CMOS camera, set at 8 output bit depth; 512 × 512 pixel ROIs were acquired with a 100× Plan Apochromatic oil immersion objective (AN 1.46). Each field was acquired with bright field illumination and double fluorescent light illumination (385/30 nm ex. for DAPI and 555/30 nm ex. for DsRed). Automatic 3D image scanning was according to the Nyquist–Shannon sampling theorem, using the inline ZEN 2.6 Nyquist Calculator. Three-dimensional scans were then processed with Zeiss ZEN 2.6 by applying the advanced Zeiss deconvolution (DCV) module. Image deconvolution was achieved by applying the constrain iterative algorithm. Spectral linear unmixing was, finally, applied to remove overlapped spectral components and background noise. Deconvolved and unmixed 3D stacks were rendered and analyzed with the ZEN 2.6 Arivis 3D module.

## 5. Conclusions

In conclusion, here we demonstrate that oxyresveratrol is a good antioxidant compound because it is able to inhibit O_2_^−^ production by human monocytes, which are strong producers of ROS. Importantly, our results indicate that a nanoparticle such as PLGA, which is generally considered to be biocompatible and harmless, could participate in the activation of oxidative events, in particular when administered in the presence of other unexpected agents, such as pathogenic fungi or their derivatives. The full characterization of the mechanisms involved in this effect went beyond the scope of the present work and will be the matter of future research. However, here we demonstrate that oxyresveratrol encapsulation into PLGA could render the nanoparticles less dangerous, mitigating the oxidative damage due to their interaction with immune cells and inhibiting an eventual synergy between the nanostructures and microbial products.

## Figures and Tables

**Table 1:** DLS data and ζ-potential of empty (PLGA), oxyresveratrol (PLGA-Oxy) and rhodamine B loaded (PLGA–Rhod) nanoparticles. The results are expressed as the mean value ± SD of three independent measures on three replica samples.

Nanoformulation	Particles Size (nm)	Polydispersity Index	Ζ-Potential (mV)
PLGA	170.2 ± 2.5	0.049 ± 0.040	−9.6 ± 0.4
PLGA-OXY	169.6 ± 3.5	0.06 ± 0.02	−7.1± 0.5
PLGA-RHOD	209.2 ± 0.6	0.057 ± 0.027	−18.2 ± 1.4

## Data Availability

No new data were created or analyzed in this study. Data sharing is not applicable to this article.

## Supplementary Materials

The following are available online, Figure S1: Emission spectra of rhodamine B-loaded PLGA nanoparticles, collected in phosphate buffer saline pH 7.4, when excited at 555 nm. The emission maximum is approximately 578 nm. Data are means of three independent measures on three replica samples. Figure S2: Calibration curve for rhodamine B in DMSO. Data are means of three independent measures on three replica samples.

## Sample Availability

Samples of the compounds are available from the authors.
