# Supplementary material for: Oxyresveratrol-Loaded PLGA Nanoparticles Inhibit Oxygen Free Radical Production by Human Monocytes: Role in Nanoparticle Biocompatibility
Source: Molecules. 2021 Jul 18;26(14):4351. doi: 10.3390/molecules26144351 (PMC8305861; doi:10.3390/molecules26144351)
Supplement: Supplementary file 1 [file molecules-26-04351-s001.zip › molecules-1298129-supplementary.pdf]

Supplementary Information for

Oxyresveratrol-loaded PLGA nanoparticles inhibit oxygen free radical production by human monocytes: role in nanoparticle biocompatibility

Marta Donini [1], Salvatore Calogero Gaglio [2], Carlo Laudanna [1], Massimiliano Perduca [2,†,*] and Stefano Dusi [1,†]

1 Department of Medicine, Section of General Pathology, University of Verona, Strada Le Grazie 8, 37134, Verona, Italy
2 Department of Biotechnology, University of Verona, Strada Le Grazie 15, 37134 Verona, Italy
* Correspondence: massimiliano.perduca@univr.it (M.P.); Tel.: +39 0458027984 (M.P)
† These authors equally contributed

Keywords: oxyresveratrol, β-glucan, PLGA nanoparticles, ROS, monocytes

Number of pages: S1-S3
Number of Figures: Figure S1-S2

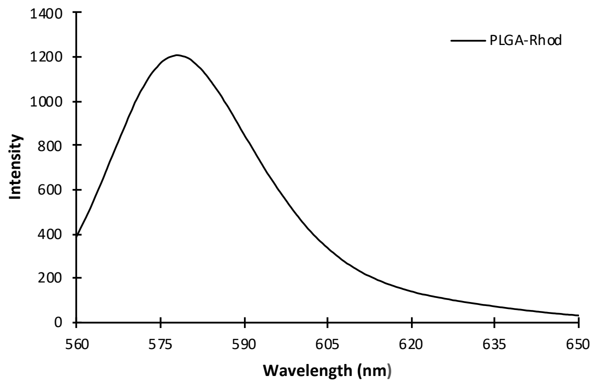

Figure S1. Emission spectra of rhodamine B-loaded PLGA nanoparticles, collected in phosphate buffer saline pH 7.4, when excited at 555 nm. The emission maximum is approximately 578 nm. Data are means of three independent measures on three replica samples.

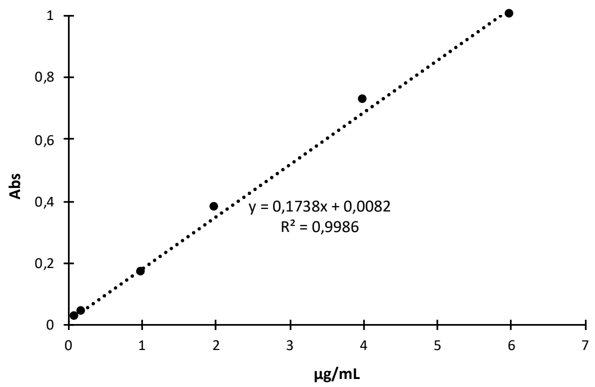

Figure S2. Calibration curve for rhodamine B in DMSO. Data are means of three independent measures on three replica samples.
